# Yoga-based lifestyle treatment and composite treatment goals in Type 2 Diabetes in a rural South Indian setup- a retrospective study

**DOI:** 10.1038/s41598-020-63133-1

**Published:** 2020-04-14

**Authors:** Geetharani Arumugam, Raghuram Nagarathna, Vijaya Majumdar, Mandeep Singh, Rambabu Srinivasalu, Rajagopal Sanjival, Venkat S. Ram, Hongasandra Ramarao Nagendra

**Affiliations:** 1 Division of Life sciences, Swami Vivekananda Yoga Anusandhana Samsathana, Bengaluru, 560106 Karnataka India; 2 Health Programme-Apollo Hospitals Ardhagiri road, Aragonda village, Tavanampalle mandal, Chittoor district, 517129 Andhrapradesh India; 3 Apollo Hospitals, Hyderabad, Telengana India

**Keywords:** Type 2 diabetes, Translational research

## Abstract

This multicentre retrospective study examined the effects of adjunct yoga-treatment in achieving composite cardiovascular goals for type 2 diabetes (T2D), set forth by the American Diabetes Association (ADA) in rural Indian settings. Records were extracted for 146 T2D patients, aged ≥20–70 years, and treated under the “Apollo Total Health Programme” for rural diabetes management, for the period April 2016 to November 2016. The study cohort comprised of two treatment groups (n = 73 each); non-yoga group (standard of care) and yoga group (adjunct yoga-treatment). Propensity score matching was applied between the study groups to define the cohort. Composite cardiovascular scores were based on the combination of individual ADA goals; A1c < 7%, blood pressure (BP) < 140/90 mmHg, stringent BP (<130/80 mmHg) and lipid, LDL-C < 100 mg/dl [risk factor for atherosclerotic cardiovascular disease]. Logistic regression was used to compare between the two treatment groups. Compared to standard of care, adjunct yoga-treatment was found to significantly facilitate the attainment of ADA composite score by 8-fold; A1c, ~2-fold; LDL-C, ~2-fold; BP < 140/90 mmHg and <130/80 mmHg by ~8-and ~6-fold respectively. This study provides the first evidence for significant efficacy of adjunct yoga-treatment for the attainment of favourable treatment goals for T2D in rural Indian settings. Clinical Trial Registration Number: CTRI/2020/02/0232790

## Introduction

Type 2 Diabetes (T2D) is a chronic progressive metabolic disease, pathophysiologically hallmarked by insulin resistance and hyperglycemia, and clinically underlined by associated severe macrovascular and microvascular ramifications^1^. According to the current estimates of the 8th edition of the atlas, International Diabetes Federation (IDF), 425 million people are afflicted by T2D across the globe^2^. As well-reflected in the current IDF estimates of 2018, with 72.95 million resident T2D populations, India is one of the most severely afflicted countries with the epidemic. Urbanization and change of lifestyle are attributed as the major underlying causes of this rising epidemic^1,3,4^.

Efficient diabetes care is a crucial aspect of the disease owing to its progressive nature^5–7^. Intensive glycemic control delays development and aids in prevention of late T2D complications^5,8^. However, glycemic control is not a stand-alone measure for efficient T2D care^6,7^. The compound pathophysiology of T2D is associated with many other deranged metabolic indices contributing to the development of cardiovascular comorbidities^1,9^. The most effective approach towards reduced mortality and morbidity in T2D appears to be comprehensive risk factor reduction targeting glycemic control, management of blood pressure and dyslipidemia^9,10^. These factors also underlie the guidelines for Standards of Medical Care issued towards efficient cardiovascular control in T2D by American Diabetes Association (ADA) which are updated annually in the month of January^7^. ADA guidelines recommend a combined target for [A1c < 7% and blood pressure (BP) < 140/90 mmHg (older cut off for BP < 130/80 mmHg) for diabetes care. Though the recent guidelines of lipid management do not specify LDL-C targets, the guidelines recommended LDL-C cut-off as <100 mg/dl for effective cardiovascular control against atherosclerotic cardiovascular disease^7^. Despite of these guidelines, at least one-third of patients with T2DM fail to achieve their ADA goals^11–13^. The ultimate success of treatment algorithms of T2D is strongly associated with concomitant synergistic lifestyle changes along with pharmacotherapy^14^. According to current ADA guidelines, lifestyle management, with medical nutrition therapy and physical activity, is a fundamental component of diabetes care^6^. These guidelines also recommend incorporation of yoga, the ancient skillset of Indian origin, into the regimen of physical activity based on individual preferences^5^. Yoga is an ancient Indian practice that emphasizes balancing of various aspects (like physical, mental, emotional, and spiritual) of an individual.

South Asian ethnicity including Indians has been characterized as one of the most challenging population for diabetes care with suboptimal status^3,14^. Though initially considered a “disease of opulence”, the recent trends suggest that diabetes has significantly impacted rural India, characterized by inadequate health care, and poverty^15,16^. The recent visibility of T2D epidemic in rural India notifies an increasing trend in the prevalence estimates; escalated from 1% to 4–10% in the year and reaching as high as 13.2% in an earlier report^16^. India is an agricultural nation, with 72.2% of the population residing in its rural sector, hence the trending high prevalence of the rural diabetes epidemic are highly alarming^16^. Several distinct cultural and socioeconomic factors define the epidemic of T2D in rural India^17^. Response and efficacy of lifestyle interventions are governed by cultural practices as well as genetic/ethnic makeup^18^. India is a vast country and an amalgamation of various social, cultural and sub-ethnic groups. In view of the reported high receptivity of yoga on diabetes management in Asians Indians^3^, we aimed to evaluate the effectiveness of this cost-effective lifestyle treatment in the marginalized rural clinical settings of Southern India.

## Results

### Baseline Characteristics

Records of a total of 146 T2D patients were retrieved. The mean age of the study cohort was 55.61 ± 10.90 years, the majority were female 64.40% (n = 94) , and 76.03% (n = 111) belonged to low socioeconomic status. The recruited study cohort had an average duration of diabetes of ~6 years. According to the ADA criteria, 54.79% (n = 80) of the total study cohort was found to be above recommended A1c targets (≥7.0%, 53 mmol/ml), 81.51% (n = 119) above BP1 targets (≥130/80 mmHg), 47.26% (n = 69), above BP2 targets (≥140/90 mmHg) and 47.94% (n = 70) above lipid targets (LDL ≥ 100 mg/dl)^19^. Overall, at baseline, 93.83% (n = 137) and 85.62% (n = 125) of the study cohort was found to be above combined ADA composite scores 1 and 2, respectively. The cohort was also observed to have generalized obesity with a mean BMI of 26.69 ± 4.58 Kg/m^2.^ Importantly, 85.62% (n = 125) of the study cohort was found to be overweight/obese according to the Asian cut off for BMI (≥23 kg/m^2^)^20^. At baseline, subjects in the yoga group had significantly lower DBP levels than the non-yoga group (yoga, 80.66 ± 9.30 mmHg vs. 84.52 ± 10.12 mmHg; P = 0.032) but were similar to the control group with respect to other parameters (Table 1).

**Table 1:** Baseline characteristics of theT2DM cohort with and without yoga treatment.

Variable	Groups		P value
	Yoga (n = 73)	Non-yoga (n = 73)	
**Age**, **Years**			
≤ 45	14 (19.18)	19 (26.03)	0.429
> 45	59 (80.82)	54 (73.97)	
**Sex**, **n (%)**			
Men	28 (38.36)	24 (32.88)	0.604
Women	45 (61.64)	49 (67.12)	
**Socio-economic status,** n (%)			
High	4 (5.48)	3 (4.11)	0.109
Medium	9 (12.33)	19 (26.03)	
Low	60 (82.19)	51 (69.86)	
**Hypertension**, **n (%)**			
Yes	25 (34.25)	29 (39.73)	0.607
No	48 (65.75)	44 (60.27)	
**Obesity**			
BMI ≤ 25Kg/m^2^	32 (43.84)	38 (52.05)	0.740
BMI > 25 Kg/m^2^	41 (56.16)	35 (47.95)	
**Known diabetes**, **n (%)**			
Yes	44 (60.27)	41 (56.16)	0.737
No	29 (39.73)	32 (43.84)	
**Duration of diabetes**, **n (%)**			
≤5 years	28 (38.36)	27 (36.99)	1.000
>5 years, n (%)	45 (61.64)	46 (63.01)	
Body mass index, BMI (kg/m^2^)	27.18 ± 4.07	26.16 ± 5.06	0.139
**Medication for T2DM**, **n (%)**			
Yes	34 (46.58)	38 (52.05)	0.508
No	39 (53.42)	35 (47.95)	
**Anti-hypertensive drugs**, **n (%)**			0.604
Yes	24 (32.88)	28 (38.36)	
No	49 (67.12)	45 (61.64)	
**Lipid-lowering drugs**			0.001
Yes	3 (4.11)	17 (23.29)	
No	70 (95.89)	56 (76.71)	
**Biochemical variables**			
A1c (%)	7.4 ± 2.3	7.7 ± 2.3	0.605
FBS, mg/Dl	100.27 ± 24.72	114.66 ± 45.042	0.189
PPBS, mg/dL	175.21 ± 46.87	197.26 ± 67.76	0.088
SBP, mm Hg	126.07 ± 13.78	130.44 ± 16.05	0.060
DBP, mm Hg	80.66 ± 9.30	84.52 ± 10.12	0.032
Total Cholesterol, mg/dl	177.71 ± 27.94	176.36 ± 34.11	0.437
Triglyceride, mg/dl	144.59 ± 28.68	143.69 ± 44.27	0.120
LDL-c, mg/dL	103.55 ± 26.53	102.16 ± 32.90	0.557
HDL-c. mg/dL	45.24 ± 1.72	45.30 ± 2.66	0.120

Test statistics, Pearson’s chi-square for categorical variables & Mann-Whitney U tests for continuous variables; FBS, fasting blood glucose; PPBS, postprandial blood glucose; SBP, systolic blood pressure; DBP, diastolic blood pressure; LDL-c low density lipoprotein cholesterol, continuous values are presented as meand ± SD.

### Effect of Yoga treatment on the attainment of ADA-laid goals

Yoga treatment was found to have a significant beneficial effect on attainment of the composite ADA goal, reflected by an increase of 2.74% [baseline, 6.85% (n = 5) to follow-up, 9.59% (n = 7)] in the number of subjects meeting the composite score 1, whereas the control group exhibited a pronounced deterioration by 4.11% [baseline (5.48%), n = 4) to follow-up (1.37%, n = 1) (Table 2)]. Similarly, there was an increase of 12.33% [baseline, 15.07% (n = 11) to follow-up, 27.40% (n = 20)] in the number of subjects meeting the composite score 2, whereas the control group exhibited a pronounced deterioration by 8.22% [baseline 13.70%, (n = 10) to follow-up 5.48%, (n = 4) (Table 2)]. When analysed by multiple regression, yoga treatment was found to be 10-fold (OR = 10.20, 95% CI = 0.69–174.19) borderline significant (P = 0.060) and ~8-fold (OR = 8.22, 95% CI = 2.02–33.49), statistically significant (P = 0.003), effective towards attaining the favourable composite ADA score 1 and 2, respectively (Table 2).

**Table 2:** Association of yoga-treatment versus non-yoga treatment with ADA cut-offs and Logistic regression at follow-up.

Outcome	Baseline			Follow up			Logistic Regression analysis		
	Yoga, n (%)	Non-Yoga, n (%)	P Value	Yoga, n (%)	Non-Yoga, n (%)	P Value	Adjusted OR	Adjusted CI	P Value
A1c target									
<7%	34(46.58)	32(43.84)	0.868	40(54.79)	27(36.99)	0.046	2.44	1.19–5.00	0.015
≥7%	39(53.42)	41(56.16)		33(45.21)	46(63.01)		1(ref)		
**BP1**									
<130/80 mm Hg	16(21.92)	11(15.07)	0.286	25(34.25)	6 (8.22)	<0.0001	6.37	2.24–18.08	0.001
≥ 130/80 mm Hg	57(78.08)	62(84.93)		48(65.75)	67(91.78)		1(ref)		
**BP2**									
<140/90 mmHg	44 (60.27)	33(45.21)	0.097	62(84.93)	29(39.73)	<0.0001	8.28	3.52–19.48	<0.0001
≥140/90 mm Hg	29 (39.73)	40(54.79)		11(15.07)	44(60.27)		1(ref)		
**LDL-c target**									
<100 mg/dl	38(52.05)	38(52.05)	1.000	40(54.79)	28(38.36)	0.068	2.22	1.06–4.68	0.035
≥ 100 mg/dl)	35(47.95)	35(47.95)		33(45.21)	45(61.64)		1(ref)		
**Composite Score 1**									
Favourable	5(6.85)	4(5.48)	0.712	7(9.59)	1(1.37)	0.013	10.20	0.69–174.19	0.060
Unfavourable	68(93.15)	69(94.52)		66(90.41)	72(98.63)		1(ref)		
**Composite Score 2**									
Favourable	11(15.07)	10(13.70)	1.000	20(27.40)	4(5.48)	0.001	8.22	2.02–33.49	0.003
Unfavourable	62(84.93)	63(86.30)		53(72.60)	69(94.52)		1(ref)		
**BMI**									
<23 Kg/m^2^	6(8.22)	15(20.55)	0.057	11(15.07)	5(6.85)	0.184	61.73	3.19–1193	0.006
≥23 Kg/m^2^	67(91.78)	58(79.45)		62(84.93)	68(93.15)		1(ref)		

OR, Odds ratio; OR (A1c target) for yoga vs. non-yoga groups, adjusted for age, sex, occupation, BMI at baseline, duration of diabetes, glycaemic indices, (FBS, PPBS and A1c) at baseline and intake of oral hypoglycaemic drug, BP, Blood pressure

OR(BP targets) for yoga vs. non-yoga groups adjusted for age, sex, duration of diabetes, socioeconomic status, SBP and DBP at baseline; and medication for blood pressure.

OR(LDL-c target) for yoga vs. non-yoga groups adjusted for age, sex, occupation, duration of diabetes, BMI, LDL-c, TG, TC and HDL-c at baseline, * additionally adjusted for lipid lowering drugs.

OR(BMI) for yoga vs. non-yoga groups adjusted for age, sex, duration of diabetes, education, smoking status and alcohol consumption, and BMI Values at baseline

Ref: Reference group, non-yoga treatment group

Composite score 1(favourable): A1c < 7.0%, BP1 < 130/80 mm Hg, LDL-c < 100 mg/dl, additionally adjusted for baseline glucose, lipid values and BMI values, and medications.

Composite Score 2 (favourable): A1c < 7.0%, BP2 < 140/90 mm Hg, LDL-c < 100 mg/dl.

With respect to the status of A1C goals of ADA (<7%), 46.58% (n = 34) subjects were found to meet criteria for in the yoga group at baseline, however, the percentage increased to 54.79% (n = 40) at follow-up (Table 2). In the non-yoga group, the percentage of subjects with A1c criteria decreased from 43.84% (n = 32) to 36.99% (n = 27) (Table 2). The difference in the distribution between the patients meeting ADA criteria for A1c was statistically significant between yoga and non-yoga treatment groups at the follow-up (P = 0.046). When analysed by multiple logistic regression, modelled by covariates, age, sex, duration of diabetes, socioeconomic status, baseline A1c values, yoga treatment was found to be significantly associated with the ~2-fold (OR = 2.44, 95% CI = 1.19–5.00, P = 0.015) higher chances of attainment of favourable A1c cut off (<7%) as compared to standard of care (Table 2).

The percentage of subjects who met the ADA-criteria with respect to favourable LDL-C, < 100 mg/dl, increased from 52.05% (n = 38) to 54.79% (n = 40) in the yoga group (Table 2). However, in the non-yoga group, there was a decrease from 52.05% (n = 38) to 38.36% (n = 28) in the number of subjects who met the LDL-C criteria (Table 2). The distribution of patients with favourable LDL-C values was not significant between yoga and non-yoga groups at the follow-up (Table 2). However, when analysed by logistic regression, adjusted for covariates and, baseline lipid status, yoga treatment was found to be significantly associated with the ~2-fold (OR = 2.22, 95% CI = 1.06–4.68, P = 0.035) increased chances for the attainment of favourable LDL-C outcome (<100 mg/dl) as compared to standard of care alone (Table 2).

We assessed the BP outcomes with old and revised favourable cut-offs recommended by ADA (Table 3). When analysed with old cut-off (<130/80 mm Hg), we could observe a pronounced increase in the percentage of subjects meeting the favourable BP outcome from 21.92% (n = 16) to 34.25% (n = 25) in the yoga group (Table 2). On the contrary, in the non-yoga group, the number of T2D patients who met BP criteria of <130/80 mm Hg decreased from 15.07% (n = 11) to 8.22% (n = 6) (Table 2). When analysed by logistic regression, yoga treatment was found to be associated with ~6.4-fold (OR = 6.37, 95% CI = 2.24–18.08, P = 0.001) increase the chances of favourable BP cut-offs (<130/80) at follow-up. When analysed with revised new BP cut-off (<140/90 mm Hg), we could observe a pronounced increase in the percentage of subjects meeting the favourable BP outcome from 60.27% (n = 44) to 84.93% (n = 62) in the yoga group (Table 2), yoga treatment was also found to be associated with 8.28-fold (95% CI, 3.52–19.48, P < 0.0001) increased chances for the revised favourable BP cut-offs,. In the non-yoga group, the number of T2D patients who met BP criteria decreased from 45.21% (n = 33) to 39.73% (n = 29) (Table 2).

**Table 3:** Distribution of continuous variables between yoga and non-yoga treatment groups at baseline and follow up.

Variables	Group	Baseline values, mean ± SE (95% CI)	Follow up values. mean ± SE (95% CI)	Mean change from baseline mean ± SE (95% CI)		Within study groups	Between study groups		
					Percentage change from baseline (95% CI)	Test statistics	Test statistics		Partial eta squared
						P value	F	P value	
A1c (%)	Yoga-group	7.40 ± 1.30 (7.03–7.77)	6.90 ± 1.29 (6.53–7.27)	−0.50 ± 1.49 (−0.15 to −0.85)	−5.03 (−9.64 to −0.42)	0.002	19.18	≤0.001	0.120
	Non-yoga group	7.75 ± 1.88	8.05 ± 1.94	0.30 ± 2.99	10.66 (1.26 to 20.1)	0.396			
Fasting plasma glucose, mg/dl	Yoga-group	100.27 ± 24.72 (93.02–107.52)	89.00 ± 10.14 (81.75–96.25)	−11.27 ± 22.91 (−16.62 to −5.93)	−8.00 (−11.7 to −4.29)	<0.0001	48.69	≤0.001	0.259
	Non-yoga group	114.66 ± 45.04 (107.41–121.91)	126.33 ± 41.03 (115.66–130.91)	11.15 ± 56.53 (−3.62 to 25.89)	18.31(5.91 to 30.7)	0.019			
Postprandial plasma glucose, mg/dl	Yoga-group	175.20 ± 46.87 (162.25–188.16)	149.70 ± 30.46 (136.74–162.66)	−25.51 ± 40.01 (−34.84 to −16.17)	−11.44 (−15.7 to −7.2)	<0.0001	49.25	≤0.001	0.262
	Non-yoga group	197.26 ± 67.76	212.53 ± 73.44	14.63 ± 117.23	19.82 (4.02 to 35.6)	0.249			
Weight, Kg	Yoga-group	68.82 ± 27.18 (66.33–71.29)	65.90 ± 10.28 (63.50–68.30)	−2.90 ± 2.37 (−3.46 to −2.36)	−4.18 (−4.96 to −3.39)	<0.0001	29.96	≤0.001	0.176
	Non-yoga group	65.82 ± 12.36	67.33 ± 11.82	1.51 ± 6.07	2.30 (−0.23 to 4.83)	<0.0001			
BMI, Kg/m^2^	Yoga-group	27.18 ± 4.07 (26.25–28.27)	26.02 ± 0.51 (25.02–27.02)	−1.14 ± 1.20 (−1.44 to −0.92)	−4.04 (−5.01 to −3.05)	<0.0001	29.01	≤0.001	0.172
	Non-yoga group	26.16 ± 5.06 (25.12–27.13)	26.92 ± 4.33 (25.79–27.88)	0.76 ± 2.67 (0.04 to 1.39)	4.06 (1.15 to 6.97)	0.002			
Systolic blood pressure, mmHg	Yoga-group	126.07 ± 13.78 (119.65–125.12)	120.77 ± 10.55 (118.03 − 123.50)	−5.30 ± 17.97 (−9.50 to −1.11)	−−3.02 (−6.22 to 0.18)	0.026	57.94	≤0.001	0.293
	Non-yoga group	130.44 ± 16.05 (128.39 − 133.86)	135.11 ± 11.88 (132.34-138.05)	4.67 ± 18.86	5.19 (1.38 to 9)	0.083			
Diastolic blood pressure, mmHg	Yoga-group	80.66 ± 9.30 (78.54-82.77)	76.08 ± 7.28 (73.97-78.20)	4.57 ± 10.51	−4.60 (−7.59 to −1.61)	0.001	40.76	≤0.001	0.225
	Non-yoga group	84.52 ± 10.11 (82.40 −86.64)	86.23 ± 9.86 (83.72-88.13)	1.71 ± 13.33 (−2.17 to 4.46)	2.94 (−0.93 to 6.81)				
Total Cholesterol, mg/dl	Yoga-group	177.71 ± 27.94 (170.60-184.82)	174.77 ± 26.87 (167.66-181.88)	−2.94 ± 33.88 (−10.85 to 4.96)	−1.49 (−2.27 to −0.708)	0.439	6.40	0.012	0.044
	Non-yoga group	176.36 ± 34.11 (169.25-183.47)	185.26 ± 28.88 (181.31-196.26)	8.90 ± 43.30 (−0.63 to 25.69)	5.05 (2.76 to 7.34)	0.055			
LDL Cholesterol, mg/dl	Yoga-group	103.55 ± 26.53 (96.74-110.36)	98.96 ± 25.42 (92.15-105.77)	−4.59 ± 33.22 (−12.34 to 3.16)	−1.03 (7.42 to 9.48)	0.312	4.56	0.035	0.032
	Non-yoga group	102.15 ± 32.69 (95.30-109.01)	108.54 ± 32.09 (102.01-116.22)	6.22 ± 48.55 (−4.44 to 20.24)	18.81 (5.71 to 31.9)	0.141			
HDL Cholesterol, mg/dl	Yoga-group	45.24 ± 1.72 (44.62-45.86)	44.54 ± 1.92 (43.92-45.16)	−0.70 ± 1.91 (−1.15 to −0.26)	−1.49 (−2.44 to −0.536)	0.013	11.11	0.001	0.074
	Non-yoga group	45.30 ± 0.32	42.79 ± 3.96	−2.51 ± 5.11	−4.72 (−7.33 to −2.11)	<0.0001			
Triglycerides, mg/dl	Yoga-group	144.59 ± 28.68 (134.25-154.93)	156.33 ± 32.05 (145.99 −166.66)	11.74 ± 31.79 (4.32 to 19.16)	31.94 (23.1 to 40.8)	0.010	27.08	≤0.001	0.016

	Non-yoga group	143.69 ± 43.96 (133.29-154.10)	211.39 ± 83.81 (174.36-195.94)	67.70 ± 93.95 (20.38 to 62.86)	59.01 (41 to 77)	<0.0001			

Data are represented as mean ± SE (95% confidence interval) *P*-value of < 0.05 was set as significant between baseline and follow-up on within group and between group comparisons; CI, confidence interval, A1c; P value- Within study groups, Test statistics t-test; P value-Between study groups test statistic- ANCOVA.

We also analysed the status of cardiovascular control for the subgroup of study cohort with uncontrolled diabetes (A1c ≥ 8.0%), n = 44. We could observe 63.16% success towards attainment of lipid goal (LDL < 100 mg/dl) and 26.32% for BP targets (130/80 mmHg) 89.47% for BP target (140/90) by 6-months of yoga treatment (data not shown). However, the controls exhibited deterioration with respect to these goals (data not shown).

Effect of yoga treatment as compared to standard of care was demonstrated with respect to the attainment of favourable BMI cut-off (<23 Kg/m^2^) for Asians (Table 2). When analysed by logistic regression, yoga treatment was found to be associated with 62-fold (OR = 61.73, 95% CI = 3.19–1193) increased chances of attainment of the favourable BMI cut-off over a period of 6 months (Table 2).

### Outcomes in continuous measures

Over the study period of around 6-months, the yoga-group exhibited significant within-group beneficial mean changes and percent changes in A1c, −0.50%, (−5.03%); FBS, −11.27 mg/dL (−8.00%); PPBS, −25.51 (−−11.44%); Wt., −2.91 (-4.18%); BMI, −1.14 Kg/m^2^ (−4.04%); SBP, −5.30 (−3.02%); DBP, −4.57 (−4.60%); TC, −2.94 mg/dl (−1.49%), HDL-c, −0.70 mg/dl (−1.49%) (Table 3). With respect to triglyceride (TG), we could observe an unexpected increase in the mean TG levels in the yoga group, 11.74 ± 3.72 mg/dl (31.94%) (Table 3). We observed pronounced worsening of the metabolic variables in the non-yoga group (Table 3). We could observe a deteriorating trend in the mean difference of these variables from baseline in the non-yoga group (Table 3). Significant within-group differences were also observed in the non-yoga group for FBS, 11.14 mg/dl (18.31%); BMI, 0.76 Kg/m^2^ (4.06%); HDL-c, −2.51 mg/dl (−4.72%), and TG, 67.70 mg/dl (59.01%). Between-study group differences between yoga group and non-yoga group very significant with respect to all the studied parameters (Table 3).

## Discussion

Type 2 diabetes is associated with vascular complications and enhanced risk of cardiovascular events. Therefore, ADA has suggested a multifactorial targeted approach towards efficient management of T2D [glycemic, lipid, and blood pressure]^5,7^. Use of such composite endpoints in clinical studies also help in the better understanding of the net effect of an intervention or a therapy rather than individual endpoints^21,22^. Further, studies targeting composite endpoints have been reported to have higher statistical efficiency as compared to those with individual endpoints^22^. We hereby highlight the grim status of diabetes management with respect to the attainment of the ADA laid primary treatment goals, in rural Indian settings. At baseline of the study, 54.79% was found to be above ADA laid A1c targets (≥7.0%), 47.26% was found to be above lipid targets (LDL-C > 100 mg/dl) and 81.51% were found to be above BP cut-offs of>130/80 mmHg, and 47.26% were found to be above the revised BP cut-offs (<140/90 mmHg)^5^. Overall, only 6.16% (n = 9) of the total study cohort was found to be meeting the composite score of all the three treatment targets at the baseline. However, a prior report by Menon *et al*.^15^, indicated only 1–3% of Indian T2D population achieving the combined treatment goals of ADA in an urban clinical setup. The pathophysiological link between obesity and T2D was also evident in the study cohort, wherein, 62.33% (n = 91) of the cohort sample was found to be obese. As majority of the cohort sample (~80%), belonged to low socioeconomic status^23^, this poor status of cardiovascular risk control could be attributed to lack of pharmacologic management, governed by poor awareness and socioeconomic status in the rural Indian settings.

Lifestyle management plays an essential role in the efficient control of diabetes status^6,24^. Yoga, as a lifestyle intervention has been reported to lead to beneficial health outcomes related to cardiovascular and metabolic disorders including T2D^25,26^. Based on its high reported receptivity and cost-effectiveness, yoga holds a strong potential as a lifestyle management skill in Indian scenario^3^. This is the first report wherein the efficacy of yoga treatment was assessed in aiding the cardiovascular fitness with respect to achievement of ADA laid primary treatment goals of T2D in rural Indian settings. Our findings reflect a magnitude of success of 10.96% attained by 6-months of yoga treatment on overweight/obese T2D Indian rural cohort. However, Ikramuddin *et al*. reported a success rate of 19% with respect to ADA composite endpoint over a period of 12 months on patients with uncontrolled T2D^27^. Further, compared to usual care, 6-months of yoga treatment was also found to be associated with 8-fold higher (OR = 8.22, 95% CI = 2.02–33.49) success towards the attainment of ADA composite scores (with revised BP cut-off of 140/90 mmHg) in rural Indian settings. With respect to individual composite goals, 6 months of yoga treatment was found to have a higher likelihood of attainment of A1c goal by ~2-fold (OR = 2.44, 95% CI = 1.19–5.00); LDL-C by ~2-fold (OR = 2.22, 95% CI = 1.06–4.68); blood pressure<140/90 mmHg by ~8-fold [OR = 8.28, 95% CI, 3.52–19.48)] in rural T2D population compared to standard of care. The observed 2-fold higher potential of yoga-treatment as compared to standard of care towards attainment of LDL-c targets in the rural Indian T2DM cohort deserves clinical attention. Control of dyslipidemia in Indian T2DM patients has been reported to be very poor; with almost half of them not reaching their LDL-C goal^28^. These findings are important as Coronary Artery Disease (CAD) mortality remains high in the Indian patients with T2DM. Similarly, the observed 8-fold increased impact of yoga on BP control as compared to standard of care is an important clinical outcome. Hypertension is a prevalent co-morbidity in T2D patients associated with an increased risk of cardiovascular events and mortality^29,30^. This coexistence has been reported to enhance the risks of nephropathy and retinopathy^31,32^.

As previously reported by Ikramuddin *et al*., intense lifestyle intervention could aid in 31% success towards attainment of glycemic, 70% for lipid and 70% for BP targets (130/80 mmHg) over a period of 12 months in a mixed ethnic population of uncontrolled T2D (HbA1c ≥ 8.0%)^27^. Interestingly, in the present subgroup of patients with uncontrolled T2D, HbA1c ≥ 8.0% yoga treatment of 6 months duration was found to be effective with 26.32% success in attainment of glycaemic control, 63.16% for lipid, and 26.32% for BP goals (130/80 mmHg). These findings indicate that if assessed for long-term effects, yoga treatment could match the magnitude of the potential of intense lifestyle-interventions as described for overweight/obese uncontrolled T2D patients^27^^.^

Glycemic control is the primary target of diabetes management strategies towards prevention of the devastating complications such as blindness, kidney failure and amputations^7,9^. We could observe significant absolute decrease in mean A1c% by 0.5 in the yoga group over a period of 6 months. The magnitude of reduction by 0.5% in A1c holds strong clinical significance, based on the reported epidemiological association between 1% reduction in the A1c value with 14% reduction in myocardial infarction (MI), 21% reduction in diabetes-related mortality and 37% reduction in microvascular complications^33^. Further significant and beneficial yoga treatment-induced mean percentage reductions in SBP (4.33%); DBP (5.66%); and TC (1.65%) could also be observed in the present study. These findings support earlier reported beneficial cardiovascular effects of yoga^25,26^.

Weight loss remains a major challenge in diabetes due to the complex interplay between metabolic, neuroendocrine and psychological factors^34^. In a prior study intensive lifestyle intervention of 1 year was reported to achieve an average 8.6% weight loss along with significant reduction of A1C, and CVD risk factors, with sustained effects up to 4 years^24^. In the present study, we could observe a 4.18% reduction in weight over a period of 6 months under yoga-treatment. Our results highlight the equivalent potential of yoga to intense weight-loss intended lifestyle interventions in overweight/obese T2D patients. A favourable and differential effect of 61-fold (OR = 61.73, 95% CI = 3.19–1193) was also observed for BMI outcome of <23 kg/m^2^ in the yoga group against standard of care. The observed significant impact of yoga-treatment on BMI outcome in diabetes bears strong clinical relevance as weight management is an important component of efficient diabetes care^7^. Further, weight loss through lifestyle changes remains the first-line therapy for T2DM^34^. Available observational evidence suggest various clinical benefits of weight loss in diabetes including improvement in glycemic control, reduced risk of cardiovascular events alongwith improvements in quality of life, mobility, and physical function^35,36^. However, we could not assess the sustenance of weight-related effects of yoga in this short-duration study. Based on the proposed self-regulation modality of yoga, wherein yoga could lead to repatterning of hedonic neurocircuitries, we speculate sustained weight-loss effects with long-term yoga-treatment^37^.

The study is limited by its observational nature. The difference-in-means method used to establish the equivalence between the study groups could be limited in its capacity to control the confounding by baseline variables. To this end, we conducted propensity score matching with “nearest neighbour” method for matching of the treatment groups for key covariates. Further, logistic regression was also done to adjust for the effect of the covariates to assess the study outcomes. The observed poor outcome in the standard of care group deserves attention of physicians and clinicians working in the rural sector of India. The poor outcome could possibly be attributed to poor adherence to medication and prescribed physical activity in the rural T2D population suggesting that there is a need to explore strategies to facilitate adherence with the patients/caregivers^17^. Since Indian patients were found to be receptive to yoga, yoga-based treatment could be a pragmatic solution for effective diabetes management. Based on the epidemic proportions of T2DM in India, there is an urgent need to conduct a large, prospective, long-term study of the efficacy of yoga on attaining all of the ADA goals in the rapidly increasing T2DM population. Early initiation of yoga treatment to target adequate diabetes care has the potential to prevent the devastating complications including not just the microvascular but also loss of time from work and quality of life.

## Methods

### Cohort identification

**Figure 1:**
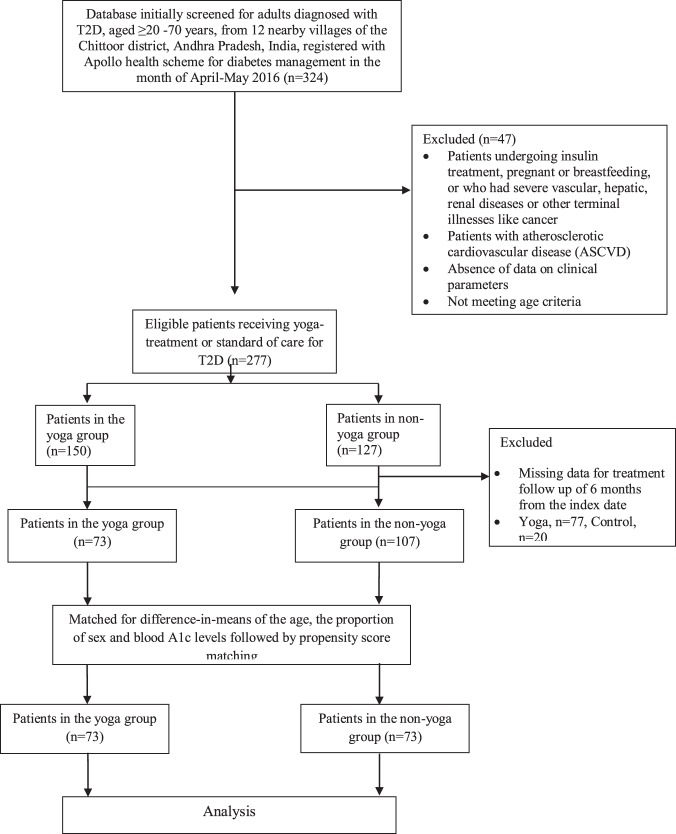
Flowchart of the study design.

The study was part of an ongoing service activity defined as the “Total Health Programme” (THP) India’s first integrated rural healthcare service delivery network, initiated by the Apollo Group (https://www.apollohospitals.com/corporate/initiatives/csr-at-apollo/total-health-programme). THP aimed at patients for various diseases across the adopted villages for effective disease management in collaboration with Swami Vivekananda Yoga Anusandhana Samsathana (S-VYASA) (http://svyasa.edu.in/). During the month of April/May 2016, 324 adults diagnosed with T2D, aged ≥20 years to 70 years, from 12 nearby villages of the Chittoor district, Andhra Pradesh, India, were originally referred to the Apollo health scheme for diabetes management. T2D was defined as per the American Diabetes Association criteria^19^. When the records were screened, out of 277 initially referred patients, only 150 were found to complete regular supervised yoga treatment, and amongst them 73 only had sufficient laboratory data until November 2016. Figure 1 details the steps involved in cohort selection. Medical records of these 73 patients were retrieved for the study. Patients undergoing insulin treatment, pregnant or breastfeeding, or who had severe vascular, hepatic, renal diseases or cancer were excluded from the study. Patients with atherosclerotic cardiovascular disease (ASCVD)^7^ were also excluded. Records of an equal number of T2D patients were retrieved as a non-yoga group who opted for only standard of care treatment at the rural Apollo clinics during the same period of time. Difference-in-means of the age, the proportion of sex and blood A1c levels were matched between yoga and non-yoga groups before finalizing the selection of the cohort sub-groups (n = 73 each). This was followed by matching the sub-groups through propensity score matching. Thus, we defined a cohort of 146 T2D patients, diagnosed with T2D as per ADA criteria^19^. Both yoga and non-yoga groups were followed from the (index date; date of first check up with prescribed treatment) until the end of 6 months. Parameters of interest were included at the time of admission/index date (baseline) and at an average of 6 months of follow-up. Written consent was obtained from the study subjects and the study was approved by the Institutional Ethics Committee of Swami Vivekananda Yoga Anusandhana Samsathana, Bengaluru, India. Informed consent was obtained from all the study subjects. All methods were performed in accordance with the relevant guidelines and regulations. The study was registered with ClinicalTrials.gov (**NCT01212133**); registration number: (CTRI/2020/02/0232790).

### Measures

The duration of yoga- treatment was approximately six months. Primary parameters of interest were the follow-up status of revised ADA laid treatment goals of diabetes;A1C < 7.0% (<53 mmol/mol), and BP cut- offs (<140/90 mmHg). Additionally based on ADA definition of risk factors of Atherosclerotic Cardiovascular Disease (ASCVD)^7^, the treatment goals also included <100 mg/dl of LDL-C, and stringent BP goals (<130/80 mmHg). Composite score was defined based on the meeting of all the target goals. Secondary outcomes were continuous measures of A1c, FBS (fasting blood glucose), PPBS (postprandial blood glucose), LDL-Cholesterol (LDL-C), SBP, DBP, weight, total cholesterol (TC), triglyceride (TG) diastolic blood pressure (DBP), and body mass index (BMI). Patient demographic and anthropometric information including age, sex, socioeconomic status, duration of diabetes, medication, weight, blood pressure, height was also extracted. BMI was calculated as weight in kilograms divided by the square of height in meters. Asian cut-off for BMI (≥25 kg/m^2^) was used to define obesity^20^.

### Intervention

The administration of yoga was carried out at Apollo rural and satellite clinics (https://www.apollohospitals.com/corporate/initiatives/csr-at-apollo/total-health-programme) for T2D patients from nearby villages. Non-yoga group, the T2D patients received the standard of care for diabetes as per ADA guidelines from a physician-coordinated team^5^. The patients were also referred for diabetes self-management education and support for strengthening and empowering their diabetes knowledge and self-care behaviors as per ADA guidelines. The yoga treatment given to the patients was derived from a validated integrated yoga module developed by Angadi *et al*.^38^. The treatment protocol included daily supervised administration of yoga sessions for one hour. The yoga module was comprised of loosening practices, asanas, pranayama, relaxation techniques, and meditation; (detailed protocol has been appended as a supplement table no. 1). Only certified yoga therapists were involved in the administration of the yoga-treatment. Both the yoga and non-yoga treatment groups were followed from the date of admission into the clinics, till November 2016.

### Statistical analyses

Missing data were minimal. Continuous variables were tested for normality with the Shapiro-Wilk test. We used descriptive statistics with mean and 95% confidence intervals [CIs]), and standard, or percentages (numbers) for representation of T2D patient’s baseline characteristics. Categorical variables were described using frequencies. Socioeconomic status was determined by using Kuppuswamy’s scale^22^. Outcome measures were compared using Analysis of covariance (ANCOVA) to adjust for baseline measures and to provide an unbiased estimate of the mean group differences. A General linear model (GLM) for multivariate analysis was developed with covariates of baseline values of the outcome variables, age, medication and duration of diabetes. P-value of < 0.05 was set as significant and < 0.0001 was set as highly significant. Statistical analysis was performed using SPSS version 21.0, Microsoft Excel-2013 and R studio version 1.1.423. For comparisons within treatment groups from baseline to follow-up, a Wilcoxon signed rank test was performed. Propensity scores were calculated for each subject based on primary baseline covariates known to be associated with diabetes treatment and/or the study outcomes, including age, sex, socioeconomic status, disease duration, medication, and biochemical parameters using the “nearest neighbour” method (Appendix, supplementary material). Logistic regression was then used to identify predictors of successful achievement of the favourable ADA and BMI outcomes. Models of the relationships were created with independent variable including age, sex, duration of diabetes, yoga treatment vs. non-yoga treatment, baseline values of variables of biological relevance.

## Supplementary information

Supplementary information.

## Data Availability

The datasets generated during and/or analysed during the current study are available from the corresponding author on reasonable request.
