# Supplementary material for: Yoga-based lifestyle treatment and composite treatment goals in Type 2 Diabetes in a rural South Indian setup- a retrospective study
Source: Sci Rep. 2020 Apr 14;10:6402. doi: 10.1038/s41598-020-63133-1 (PMC7156497; doi:10.1038/s41598-020-63133-1)
Supplement: Supplementary file 1 — Supplementary information. [file 41598_2020_63133_MOESM1_ESM.docx]

**Yoga-based lifestyle treatment and composite treatment goals in Type 2 Diabetes in a rural South Indian setup- a retrospective study**

Geetharani A^1^, Nagarathna R^1^, Vijaya M^1*^, Mandeep S^2^, Rambabu S^2^, Rajagopal S^2^, Venkat R^3^, Nagendra HR^1^

**Supplementary Table 1: Detailed description of the yoga module**

| **SNo** | **Name of the practice** | **Duration** |
| --- | --- | --- |
| **1** | **Starting Prayer** | 2 mins |
| **2** | **Preparatory Sukshma Vyayamas and Shithililarana Practices (loosening practices)**   - Urdhvahastashvasan (Hand Stretch Breathing 3 rounds at 90°, 135°, 180° each) - Kati-Shakti Vikasaka (3 rounds each)   Forward and Backward Bending **and t**wisting   - Sarvangapushti (3 rounds clockwise, 3 rounds anti-clockwise) | 1. mins |
| **3** | **Surya Namaskara (sun salutation)**   - 10 step fast Suryanamaskara 6 rounds - 12 step slow Suryanamaskara 1 round - (To be avoided by those with knee pain, cardiac problems, renal problem, low back pain, retinopathy and the elderly who are weak and not flexible; instead they can do Chair SN) Modified version Chair SN: 7 rounds | 9 mins |
| **4** | - **Asanas (postures)** (1 minute per asana)   **Standing (1 minute per asana)**   - Trikonasana (triangle pose), Pravritta Trikonasana (revolved triangle pose), Prasarita Padhastasana (intense leg stretch)   **Supine postures**   - Jatara Parivartanasana (abdominal twist pose), Pavanamuktasana (Wind-relieving pose), Viparitakarani (inverted lake pose)   **Prone**   - Bhujanagasana (cobra pose), Dhaurasana (bow pose) followed by Pavanmuktasana   **Sitting**   - Mandukasana (Frog pose), Vakrasana /Ardhamatsyendrasana (Spine twisting posture), Paschimatanasana (seated forward bending), Ardha Ushtrasana (half camel pose)   At the end, relaxation with abdominal breathing in supine position (vishranti), 10-15 rounds (2 minutes) | 15 mins |
| **5** | **Kriya**   - Agnisara: 1 minute **\|** - Kapalabharti   (60 breaths per minute for 1 minute followed by rest for 1 minute) | 3 mins |
| **6** | **Pranayama**   - Nadishuddhi (for 6 minutes, with antarkumbhaka and jalandhar bandha for 2 sec) - Bhramari (3 minutes) | 9 mins |
| **7** | **Meditation** (For stress management for deep relaxation and silencing the mind) | 15 mins |
| **8** | **Resolve** ( | 1 min |
| **9** | **Closing Prayer:** Sarvebhavantu Sukhinaha… | 1 min |
|  | **Total** | **60 mins** |

The integrated module consisted of cleansing techniques, yogic postures (asanas), breathing practices (pranayama) and meditation. Based on American College of Sports Medicine (ACSM) and the American Heart Association (AHA), majority of the yoga asanas are classified as light-intensity exercise. Most of these stretching exercises with standing, supine, prone and sitting postures have been reported to enhance cardiorespiratory fitness. Cardiorespiratory fitness is a strong biological index of physical activity and its reduced levels are reported as reliable risk marker for diabetes.

Surya Namaskar (sun salutations) Paschimatanasana have been reported to elicit metabolic equivalent intensities (>3) in the moderate-intensity aerobic range. Yoga is not typically practiced at an intensity that meets the ACSM/AHA recommendations for moderate-intensity aerobic exercise

Nadishuddhi, cleansing of nadis that refer to fine channels that carry life force; Bhramari, humming bee breath named after the humming sound produced at the back of the throat while practice, have shown to exert parasympathetic dominance, serves as a breathing as well as relaxation techniques

Cleansing techniques such as Kapalbharthi and Agnisara Kriya, are forceful breathing techniques. Agnisara is derived from Hathayoga that consists of alternate, forceful retractions and protrusions of abdominal wall, executed over a period of 20-20s of apnoea, involves repeated and long-lasting stimulation of visceral and somatic receptors. Kapalbharathi consists of forceful contraction of abdominal muscles, permits rapid exhalation and inhalation through nostrils.

**Propensity scores**

**
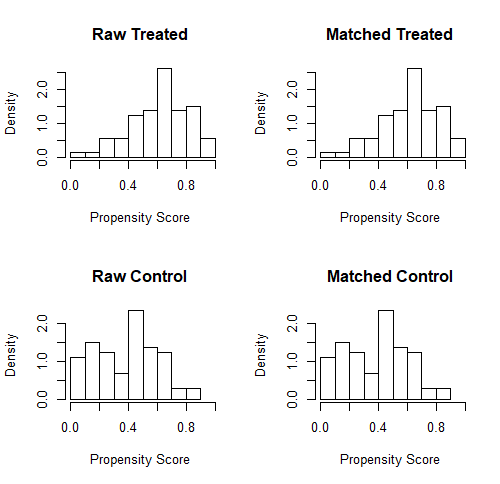

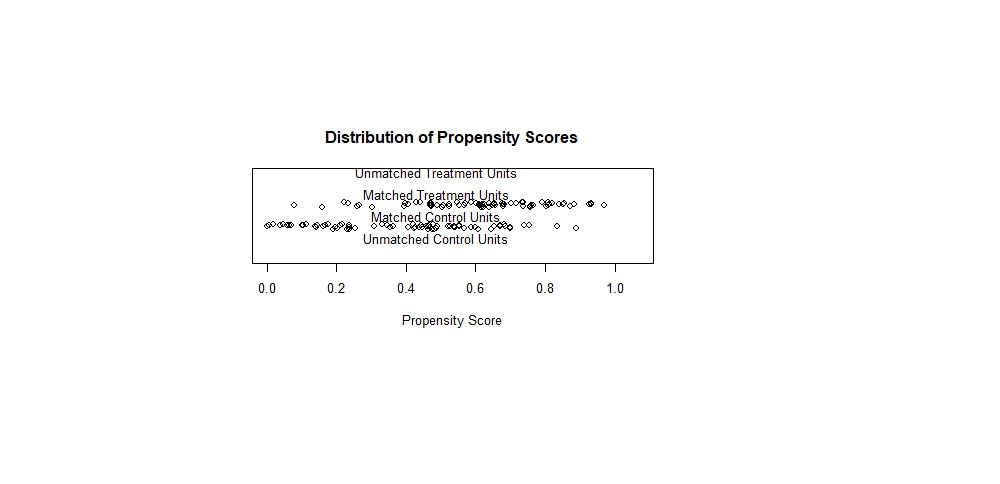
**

**Results of summary of balance of all and matched data**

| **Call:** |
| --- |
| matchit(formula = Group2 ~ Age + Gender + Lipidloweringdrugs + |
| MedicationDM + FBS1 + PPBS1 + HbA1c + Total.cholesterol.1 + |
| TGL1 + HDL1 + Idlc1 + Systolic1 + Diastolic1 + BMI1 + SE_Apollo + |
| DurationDM, data = mydata, method = "nearest", ratio = 1) |
|  |
| Summary of balance for all data: |
| Means Treated Means Control SD Control Mean Diff eQQ Med eQQ Mean eQQ Max |
| distance 0.6136 0.3864 0.2253 0.2272 0.2104 0.2272 0.3311 |
| Age 55.7260 53.6301 11.2392 2.0959 2.0000 2.5890 7.0000 |
| Gender 1.6164 1.6712 0.4730 -0.0548 0.0000 0.0548 1.0000 |
| Lipidloweringdrugs 0.0411 0.2329 0.4256 -0.1918 0.0000 0.1918 1.0000 |
| MedicationDM 0.4658 0.5205 0.5030 -0.0548 0.0000 0.0548 1.0000 |
| FBS1 100.2740 114.6575 45.0423 -14.3836 5.0000 15.4521 76.0000 |
| PPBS1 175.2055 197.2603 67.7582 -22.0548 15.0000 23.5342 74.0000 |
| HbA1c 7.4014 7.7479 1.8819 -0.3466 0.4000 0.5110 1.9000 |
| Total.cholesterol.1 177.7123 176.3562 34.1090 1.3562 4.0000 5.9041 58.0000 |
| TGL1 144.5890 143.6986 43.9626 0.8904 16.0000 18.5342 139.0000 |
| HDL1 45.2447 45.2942 2.6380 -0.0496 0.9600 1.1082 8.3400 |
| Idlc1 103.5499 102.1551 32.6671 1.3948 2.7200 4.5942 56.8800 |
| Systolic1 126.0685 130.4384 16.0554 -4.3699 6.0000 5.3836 10.0000 |
| Diastolic1 80.6575 84.5205 10.1175 -3.8630 4.0000 3.8630 13.0000 |
| BMI1 27.1781 26.1644 5.0635 1.0137 1.0000 1.2603 6.0000 |
| SE_Apollo 2.7671 2.6575 0.5583 0.1096 0.0000 0.1370 1.0000 |
| DurationDM 7.3151 6.1370 4.1039 1.1781 0.0000 1.3973 20.0000 |
|  |
|  |
| **Summary of balance for matched data:** |
| Means Treated Means Control SD Control Mean Diff eQQ Med eQQ Mean eQQ Max |
| distance 0.6136 0.3864 0.2253 0.2272 0.2104 0.2272 0.3311 |
| Age 55.7260 53.6301 11.2392 2.0959 2.0000 2.5890 7.0000 |
| Gender 1.6164 1.6712 0.4730 -0.0548 0.0000 0.0548 1.0000 |
| Lipidloweringdrugs 0.0411 0.2329 0.4256 -0.1918 0.0000 0.1918 1.0000 |
| MedicationDM 0.4658 0.5205 0.5030 -0.0548 0.0000 0.0548 1.0000 |
| FBS1 100.2740 114.6575 45.0423 -14.3836 5.0000 15.4521 76.0000 |
| PPBS1 175.2055 197.2603 67.7582 -22.0548 15.0000 23.5342 74.0000 |
| HbA1c 7.4014 7.7479 1.8819 -0.3466 0.4000 0.5110 1.9000 |
| Total.cholesterol.1 177.7123 176.3562 34.1090 1.3562 4.0000 5.9041 58.0000 |
| TGL1 144.5890 143.6986 43.9626 0.8904 16.0000 18.5342 139.0000 |
| HDL1 45.2447 45.2942 2.6380 -0.0496 0.9600 1.1082 8.3400 |
| Idlc1 103.5499 102.1551 32.6671 1.3948 2.7200 4.5942 56.8800 |
| Systolic1 126.0685 130.4384 16.0554 -4.3699 6.0000 5.3836 10.0000 |
| Diastolic1 80.6575 84.5205 10.1175 -3.8630 4.0000 3.8630 13.0000 |
| BMI1 27.1781 26.1644 5.0635 1.0137 1.0000 1.2603 6.0000 |
| SE_Apollo 2.7671 2.6575 0.5583 0.1096 0.0000 0.1370 1.0000 |
| DurationDM 7.3151 6.1370 4.1039 1.1781 0.0000 1.3973 20.0000 |

| **Percent Balance Improvement:** |  |  |  |
| --- | --- | --- | --- |
| Mean Diff. eQQ Med eQQ Mean eQQ Max | | | |
| distance 0 0 0 0 | | | |
| Age 0 0 0 0 | | | |
| Gender 0 0 0 0 | | | |
| Lipidloweringdrugs 0 0 0 0 | | | |
| MedicationDM 0 0 0 0 | | | |
| FBS1 0 0 0 0 | | | |
| PPBS1 0 0 0 0 | | | |
| HbA1c 0 0 0 0 | | | |
| Total.cholesterol.1 0 0 0 0 | | | |
| TGL1 0 0 0 0 | | | |
| HDL1 0 0 0 0 | | | |
| Idlc1 0 0 0 0 | | | |
| Systolic1 0 0 0 0 | | | |
| Diastolic1 0 0 0 0 | | | |
| BMI1 0 0 0 0 | | | |
| SE_Apollo 0 0 0 0 | | | |

**Sample sizes:**

**Control Treated**

**All 73 73**

**Matched 73 73**

**Unmatched 0 0**

**Discarded 0 0**
